# Unsettling the fluidity of practice and dealing with threat: the experiences of paediatric pharmacists in response to the admission of adult COVID-19 patients requiring intensive care in a paediatric tertiary hospital

**DOI:** 10.1093/ijpp/riac074

**Published:** 2022-10-28

**Authors:** Bernie Carter

**Affiliations:** Faculty of Health, Social Care and Medicine, Edge Hill University, Ormskirk, UK

**Keywords:** Compassion-focused therapy, paediatric pharmacist, COVID-19, intensive care, well-being, clinical psychology

## Abstract

**Objectives:**

The COVID-19 pandemic impacted the lives of pharmacists, resulting in new ways of working. Little literature focuses on the experiences and well-being of hospital pharmacists, particularly on paediatric pharmacists. The setting – a paediatric stand-alone tertiary hospital – opened to adult ICU COVID-19 patients for two time periods. Paediatric pharmacists had to shift their roles; this impacted their well-being. Paediatric ICU clinical psychologists provided support using a compassion-focused therapy (CFT) model to guide thinking, reflection and promoting behaviour change. This study aimed to explore the experiences and perceptions of the paediatric pharmacists working in a paediatric stand-alone tertiary hospital before, during and after the admission of adult COVID-19 patients into ICU and their experiences of support offered by clinical psychologists.

**Methods:**

A qualitative interpretative design using remote photo-elicitation interviews was adopted. Data analysis was undertaken using the six stages of reflexive thematic analysis.

**Key findings:**

Five paediatric pharmacists participated; four deployed to work in the A-ICU (from PICU) and one deployed to work in the PICU (from ward-based work). An overarching theme, ‘Unsettling the fluidity of practice and dealing with threat’, is supported by four key themes ‘Context and preparation’, ‘Dread and challenges’, ‘Keeping it together’ and ‘Lessons learned’. The fluidity of the pharmacists’ practice was unsettled as they dealt with the threats and sought resources (drive) to enable optimal care delivery. Soothe techniques helped compensate for threats, and promote resilience and well-being.

**Conclusion:**

The CFT model has been useful in the longer term with the adoption of a more open, compassionate approach to their work and colleagues.

## Introduction

Paediatric clinical pharmacists’ role encompasses the assessment of the child, safe administration and optimisation of their medication(s), and evaluating the appropriateness and effectiveness of the child’s medications.^[1]^ Skills and experience within specific settings result in pharmacists becoming confident in their knowledge and decision making and their practice becoming informed, fluid and accomplished. However, that fluidity of practice can be challenged if new and exceptional circumstances occur.

The coronavirus pandemic (COVID-19)^[2]^ has required all healthcare staff, including pharmacists, to adapt practice and there is evidence of the breadth of impact and contribution by pharmacists.^[3–7]^ Most research addressing COVID-19 within adult intensive care unit (ICU) has focused on the vital roles of, and personal and professional impacts, on doctors and nurses.^[8–10]^ Less work specifically addresses pharmacists’ roles within adult COVID-19 ICU settings.^[5,11,12]^ No literature considers the extreme demands placed on paediatric pharmacists required to adapt to delivering care to adult ICU COVID-19 patients.

Within the UK, the extreme pressures for ICU beds to deal with peaks of the sickest COVID-19 patients resulted in many measures; one response, that created unique and profound challenges, was opening an adult ICU (A-ICU) within a standalone paediatric tertiary hospital. This hospital opened to adult ICU COVID-19 patients for two time periods: March 2020–May 2020 (11 patients) and December 2021–March 2022 (10 patients). Within this setting, the strategy was to mobilise paediatric ICU (PICU) pharmacists to the new A-ICU and paediatric ward-based pharmacists to deliver care in PICU. In both situations, the paediatric pharmacists were confronting new and unfamiliar situations and patient populations and had to adapt and develop their knowledge and skills

The requirement to acquire new knowledge and skills under a particularly uncertain and shifting set of demands, along with the fear and uncertainty of the pandemic and lockdown created huge professional and personal pressures. They reached out and accessed support from the established PICU clinical psychologists whose role encompasses support for children, families and staff. Regular, remote weekly, then fortnightly meetings offered and continue to offer the opportunity to share and process experiences. These meetings were underpinned by an approach based on the compassion-focused therapy (CFT) model which provides a framework to understand why people may hold fears and concerns, the impact of these feelings and how people can manage their emotions.^[13]^ It is based on three affect systems: threat defence (focused on detecting threats and defensive emotions such as anxiety and defensive behaviours such as fight and flight); drive (resource seeking) and soothe/contentment (associated with feelings of well-being).^[13]^ CFT has been used with children and adults and in many contexts such as grief therapy and trauma therapy.^[14–16]^

The overall aims of this qualitative study were to (1) explore the experiences and perceptions of the paediatric pharmacists working in a paediatric stand-alone tertiary hospital to the shift in their roles due to the admission of adult COVID-19 patients requiring intensive care and (2) their experiences of the support offered by clinical psychologists.

## Methods

### Study design

The study adopted a qualitative interpretative approach. The study was conducted in accordance with Consolidated Criteria for Reporting Qualitative Research (COREQ) guidelines.^[17]^ Ethics approval (ETH2021-0077); FoHSC&M Ethics Committee, Edge Hill University.

### Target population

The target population was paediatric pharmacists (*n* = 9) working in the paediatric tertiary setting whose roles shifted as they prepared for the admission of and step down from adult COVID-19 patients (March 2020–May 2020; December 2021–March 2022). Of the nine paediatric pharmacists, six experienced PICU pharmacists were deployed to A-ICU, three ward-based pharmacists were deployed to work in PICU, and one pharmacist was deployed to work in the high dependency unit. One clinical psychologist (CP) participated to provide context about their role and responsibilities. Invitations (letter of invitation, study information sheet, and consent checklist) were sent by email by a nominated pharmacist.

### Procedure

Individual remote participant-driven photo-elicitation (PE) interviews^[18,19]^ were undertaken (June–August 2021) via Microsoft Teams using video mode at a mutually convenient time in settings (home or workplace) which offered appropriate levels of privacy. Verbal consent was gained before the interview started; a copy of the completed consent form was emailed to the participant.

PE is a visual research approach which uses photographs or other visual images as triggers to generate verbal discussion.^[18,19]^ In this study, PE aimed to create the potential for sensitive, participant-led dialogue.^[20]^ PE has been used to explore experiences and impacts related to the COVID-19 pandemic in patients and healthcare staff.^[21,22]^ To date, no PE studies have been identified that have involved pharmacists.

Participants were invited to create or locate and share one or more photographs or other visual image(s) that they perceived as being relevant to their experiences of PICU/A-ICU during the pandemic. If a participant did not wish to share an image, they could share a ‘prose image’ (a verbal description of an image). Participants shared copies of their image(s) with the researcher (BC) and selected the image they wanted to start with and moved to other images as the interview progressed. The interviewer asked an opening question ‘please tell me about this image and why you chose it’ and then engaged in a dialogue with the participant to explore their experiences and perceptions of their practice, the challenges (emotional and professional), the impact their experiences had on them. Questions were also asked about the support they had received or had given to their colleagues and how this support helped them navigate and manage the challenges they faced during this difficult time. If needed, additional questions were asked to prompt discussion (Supplementary File S1). At the end of each interview, the researcher checked if the participant was ‘OK’ and sent them a ‘Thank you and helpful information’ sheet (Supplementary File S2) that reminded them to seek emotional support, as needed.

### Data analysis

All interviews were transcribed verbatim and analysed (no software used) using the six stages of reflexive thematic analysis (familiarisation, generating initial codes, constructing themes, reviewing themes, defining and naming themes and producing a report) allowing the shift from descriptive to interpretative analysis.^[23,24]^ A key component of reflexive thematic analysis is positioning the researcher. The research was conducted by BC (woman, PhD, academic children’s nurse). Although she no longer has a clinical role, her clinical experience was in children’s intensive care, so there was an instinctive lived connection with the setting and context. She is familiar with and has strong research connections with the hospital and has visited (before and unconnected with the study) the units in which the practice described in the study occurred.

Due to the sample size and potential to identify participants, participant labels have not been assigned to illustrate quotes and photographs have not been included (this aligns with conditions of approval from ethics review). Three participants reviewed the final themes.

## Results

Five paediatric pharmacists participated; four deployed to work in A-ICU (from PICU) and one from the wards to work in PICU. One CP participated. Six potential participants were either unavailable or did not respond to the invitation. Interviews lasted 30–90 min (average 60 min). For PE triggers see Table 1. The findings from the CP’s interview are summarised separately, as a condensed narrative (Table 2). Additional illustrative quotations (Table 3) are referred to in the text as Additional Quote 1 (AQ1), etc.

**Table 1:** Overview of photo-elicitation materials

Photo-elicitation triggers	Photographs (*n*=12) shared by four participants.
	Prose-image (*n*=1) shared by one participant.
	Object (*n*=1) shared by one participant.
Images shared	Photographs (*n*=4) depicting either themselves or other people wearing full PPE.
	Photographs (*n*=3) portraying the six (A-ICU) pharmacists taken in front of a rainbow mural. Photograph (*n*=1) portraying a news image of an adult patient in ICU.
	Photograph (*n*=1) portraying a Zoom call with their family.
	Photograph (*n*=1) portraying a hilly road.
	Photograph (*n*=1) portraying ‘The Scream’ by Munch.
	Photograph (*n*=1) portraying an image of arrows representing the disconnect between Britain and Europe.

**Table 2:** The clinical psychologist’s story

‘It was just by chance that one of the pharmacists had a conversation with us. We’d said “We’re open, we’re offering support … maybe it would be a helpful thing to offer the pharmacists a separate space to come to talk about their own individual experiences”.
Initially that we were just trying to explain who we were, what we were about, what we were trying to do, what we could offer. There was clearly a need, but I got a sense they perhaps didn’t know what needed or what they wanted. All they really knew was they were finding things tough, and we may be in a position to be able to offer something to be able to help with that.
It was a big commitment in time [for us]. One [of us] would lead the facilitation whilst the other psychologist just kind of kept an eye on what was happening in the group, kind of picked up on things that perhaps the facilitator wouldn’t necessarily pick up on and we took it in turns to do that. There was always that comfort in knowing there was just another person keeping an eye on the team.
We would start with just going round, just going round asking people just say a few words about how things feel for you today and it felt very early on that a lot of what people were talking about fitted quite nicely in the compassion focused therapy model. And so, we started to slowly introduce that model and they just, they very, very quickly just connected with it. It’s a very visual model so we would just literally get the three circles on the screen and straight away they said “that’s exactly how we feel”. So, because it seemed to work so well, we just kept with that. We used that model every week…it was a very good tool to just start the conversation and almost to set the tone and structure the session.
We often run groups where actually it’s incredibly difficult to engage the people, start conversations and allow narratives and themes to grow and emerge. It never felt hard with the pharmacy group. I knew that we would be able to offer something that would be of some value. We got feedback from them that we were doing something [good] and that really helped me because at a time when you’re feeling completely helpless and to feel yes, we’re making a difference was something that I guess I probably needed at that time too. It was incredibly difficult witnessing their struggles and distress…I had a sense of what they were going through…I was on some level dealing with it myself, I was struggling to feel containment myself.
Resilience is one of those words that’s just branded around … initially for me it kind of implied that if you’re not resilient then you’re weak and I didn’t like the dichotomy. And yet it is probably a word that I would start to use more now in relation to myself…[to help explain] how did we manage to do what we did’.

**Table 3:** Additional illustrative quotations (AQs)

**Context and preparation**		
Preparing for change	AQ1	‘[it was] less of a healthcare challenge for us [hospital]’ as ‘in a weird way we were fairly protected [from Covid-19] for a while’.
	AQ2	‘we were inexperienced critical care pharmacists, left on our own a bit because our colleagues were sorting out the adults’.
Changing mindsets	AQ3	‘we gave ourselves a huge amount of work trying to do in a couple of weeks what adult hospitals had developed through years and years of practice’.
	AQ4	‘we kind of look at things per dose, we like to see ceiling doses, maximum doses so we can individualise the treatment to our patients because the paediatric population is so ever changing in weight, height and so on, whereas they [adult pharmacists] tend to be more restrictive [in their approach to dosing]’.
	AQ5	‘[writing SOPS for] how we would authorise sharing vials which previously we would say you were never allowed to do that…but obviously were necessary in this emergency situation’.
**Dread and challenges**		
Facing the threat and reality	AQ6	‘[the] physical checking and re-checking [to ensure]’ ‘PPE was donned correctly’… ‘all of a sudden having to wear two gloves then people were tying Sellotape to the end of the gown to cover the gloves and then putting gloves on top…wearing welly boots instead of the clogs’.
	AQ7a	‘…it was an assault to the senses …lots of people, …lots of noise, it just felt busy, noisy, claustrophobic, … There was a moment where I just kind of had to tell myself “Okay you’ve got this, just breathe”’.
	AQ7b	‘that feeling and that thought and the noises and the smells and yeah, the chaos – you can smell it or feel it sometimes in a room – I just recall it very vividly. I just remember walking towards where the noise was coming from and the beeps were coming from and the rustling of PPE. And yeah, just the dread and fear, yeah’.
	AQ7c	‘I’ve never had a panic attack or anything like that in my life but I remember that first time walking on and … I wasn’t hyperventilating but I was, my breathing was really high. My heart was beating out of my chest and it took me a good five or ten minutes just to, I was standing there with a prescription with my pen and I just didn’t do anything, I was like …[deep breathing]… trying to just get myself, which is ridiculous. …It really was so hard’.
	AQ8	‘…we were full on in threat mode and it was threat in work and threat outside of work… threat to your loved ones’.
	AQ9	‘we were asked to take just ten patients and it was only going to be for four weeks... I know I just said just ten patients but that’s ten patients, each patient is not a number, it’s a loved one of somebody. That meant that those ten patients were treated when otherwise they might not have been’.
Unsettling practice and confidence	AQ10	‘[initially] I don’t think we really had a grip on things and the doses, the doses are worked out differently, …the basic principles might be the same a lot of the medications in adult care [but I] didn’t really feel I had much of a clue about [things to start with]’.
	AQ11	‘my mentality towards [my] first adult patient was, I just need to make sure this person doesn’t die because of something I’ve missed…make sure that they don’t get an overdose in a drug or they don’t get an interactive drug that will kill them or they don’t get something that will cause a side effect that will kill them’.
	AQ12	‘ten adults took you double maybe triple the amount of time it would take you to do ten children because of the lack of familiarity and the lack of understanding of the disease that was forming in front of us … It never felt quick, it always felt long [maybe] because of our [checklist type] strategy] for each patient – review the patient, review the prescription, make sure the stocks were up to date so the communication was right around stock, deal with any other issues which might crop up…’.
	AQ13	‘it’s like being a Band 6 pharmacist again, what’s this for, what’s the dose, what are the drug interactions, what are the side effects, [on what] kind of patients should it be avoided’.
**Keeping it together – giving and getting support**		
Reaching for support	AQ14	‘we identified it that a lot of the conversations …were way beyond the skills of anyone in the room to talk through and break down, we felt as if it was beyond our expertise to facilitate so that’s when we got the help’.
	AQ15	‘They were fantastic. I’ve never had anything to do with psychologists on a personal level… never had any counselling or anything like that. I was probably a bit sceptical about how’s it going to help us having the clinical psychologist. I didn’t really know what they did, so it was just really lovely to see what a benefit they were to us’.
	AQ16	‘made us feel like they understood that our fears were valid, that we were doing a good job and we were in a situation where we were doing the best we could and that was fine’.
Engaging a compassionate mindset	AQ17	‘our threat bubbles were so large and our drive bubbles were so large but none of us were soothing; none of us were looking after ourselves’.
	AQ18	‘threat mode which is kind of like when you’re anxious, you’re worried… the fright if you like…and the drive mode which is…the lack of knowledge and it’s like “oh my god, I need to understand this, I need to learn this, if I don’t learn this then I can’t do my job”’.
	AQ19	‘[they] gave us tools and coping mechanisms to work with… introduced us to calming techniques… helped us find ways to compensate for the excess levels of fear and threat and anxiety we were experiencing by doing …or focusing on other things’.
	AQ20	‘I think probably saved me from being possibly having more serious [wellbeing problems] down the line… that space and time where I could share… [was important]’.
	AQ21	‘I guess it wasn’t for everybody. Maybe some people like to deal with things in a different way’.
**Lessons learned**		
You’re part of a wider team	AQ22	‘there was a definite camaraderie about [everyone] being in it together…everybody respected everybody else…it was very stressful, but on both occasions I think we adapted and worked well as a team and did a really good job actually, you know under tricky circumstances’.
You’re not indestructible	AQ23	‘it disarmed and destroyed all the barriers built through my professional life on how you deal with grief and anger and frustration’.
You’re better with compassion	AQ24	‘maintaining a mindfulness approach, [taking] 30 seconds to calm down and ‘drop the anchor’” and “trying to do things that compensate [and] switching off at home’.
	AQ25	‘if something’s bothering you, please don’t feel afraid to talk about things….being open about things affecting us and kind of not shying away, we’re human at the end of the day’.
	AQ26	‘before [in one-to-ones] we’d just get on with the job, before I didn’t necessarily want to go there [difficult places] and didn’t think they’d want to talk about it. I think now I’m much more prepared to say “So are you really ok, come on then, how are things”?’

**Figure 1:**
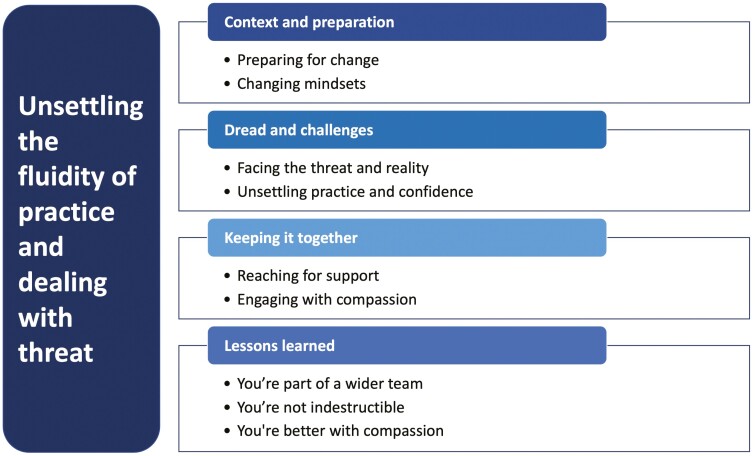
Overview of themes.

One overarching theme – unsettling the fluidity of practice and four sub-themes were identified Figure 1.

### Context and preparation

This theme primarily addresses the time before the first adult patient was admitted in March 2020 during a time of high level of national and international uncertainty about the pandemic. It addresses the unique position of this stand-alone tertiary hospital, the logistical and information-related preparations and the changes to mindsets.

#### Preparing for change

Initially, as seen in other paediatric hospitals they were relatively ‘spared’ and protected from the full impact of COVID-19 compared to adult healthcare settings (AQ1). However, once preparation commenced for admitting adult COVID-19 patients to A-ICU, the impact deepened. The decision to move ‘*experienced paediatric critical care pharmacists*’, to A-ICU and ward pharmacists to PICU was acknowledged as ‘*throwing [everyone] in at the deep end*’ (AQ2).

Preparation involved formulating a structured approach by *‘taking a piece of this and that idea’* and asking colleagues in Europe and adult centres *‘for information…on doses, how to monitor side effects etc.’* This reaching out for information occurred at a time when adult colleagues had *‘diminished their capacity to respond’* as they were *‘completely overwhelmed’.*

They had to quickly identify and create robust information to support practice (AQ3), and undertake ‘*logistical preparation, back up rotas, etc.’* and manage the practicalities of ‘*stock shortages for critical care drugs’.*

#### Changing mindsets

There was a perception that *‘adult and paediatric practice, differ in the way we see problems and approach problem solving’* such as adult dosing tends to be based on *‘this is the dose, this is the range’.* This more regimented thinking was far different to the nuanced, individualised dosing decisions in paediatric practice where a *‘one-size-fits’* approach does not work (AQ4). This tension resulted in the pharmacists making dosing decisions and creating guidelines, reflecting their own ways of working. New standard operating procedures, such as authorising vial sharing (AQ5), were created that were at odds with usual practices.

### Dread and challenges

This theme addresses the sense of dread about the threat of COVID-19 as well as the fear associated with having to step up to either A-ICU or PICU and the challenge to ensure their practice was the best it could be.

#### Facing the threat and reality

Adult COVID-19 patients represented a real threat for the pharmacists working in A-ICU as there were concerns about maintaining personal safety and protecting their family, *‘I didn’t want to infect myself, or bring it home’*. Early on the virus was perceived as being ‘*like Ebola, one droplet…you’re going to die’*. The wearing of personal protective equipment (PPE) was stressful as *‘it was hot…stuffy, you couldn’t communicate well with people’.* Pharmacists worried about the consequences of incorrectly donning on/off their PPE and were stressed when guidance changed (AQ6).

The first time they stepped into A-ICU in full PPE evoked profound, *‘all-consuming’* emotional responses; a sense of shock and threat that will *‘stay forever’* (AQ7a, AQ7b, AQ7c, AQ8). Even during the second time period, the emotional threats remained, perhaps exacerbated by the fact that they were already drained by the pandemic. Despite these threats *‘even on the toughest days’* the A-ICU pharmacists *‘still wanted to be there’* (AQ9). The PICU pharmacist was not exposed to the same close-up physical threat of being in a COVID-19 environment and they were not required to wear full PPE in PICU. Thus, their experience was qualitatively different in this respect and more reflective of the way in which paediatric hospitals were relatively ‘spared’ the direct intensity of threat within adult settings.

#### Unsettling practice and confidence

A core challenge, particularly for the A-ICU pharmacists, was their length of time *‘out of touch with adult practice’* (average 15 years), resulting in feelings of ‘*discomfort’, ‘inadequacy’* and *‘everything feeling very alien’* (AQ10). Part of this related to unfamiliarity with the co-morbidities that adult patients were likely to co-present with, for example, ‘*chronic [adult] heart disease, prostate [disease], and “adult” cancers’*. Another challenge was the weight difference between PICU patients (anything from 3 to 50 kg) and A-ICU patients (100–120 kg). Typically, the paediatric practice uses *‘weight-based dosing’* and *‘micrograms per kilo per hour’* but *‘it’s millilitres per hour in adults’*; adults could *‘use as much that we would use in the whole ward in one day normally’.*

All pharmacists, especially the A-ICU pharmacists, talked of how their usual goal of medicine optimisation was constrained by a lack of familiarity with drugs, dosages, multi-co-morbidities and potential adverse effects. Initially, they adopted a more limited goal (practising safely), summed up as ensuring *‘the person doesn’t die because of something I missed’* (AQ11). Furthermore, the complexity of A-ICU meant that prescription reviews, especially during the first time period, took *‘way longer’* due to *‘checking, re-checking and checking decision again’* rather than relying on the expertise and embodied knowledge (AQ12). On PICU, reviews also took longer due to inexperience (AQ13).

### Keeping it together

This theme presents aspects of support needed and support given during both times when adult patients were being cared for.

#### Reaching for support

Most conversations focused on aspects of emotional stress. The A-ICU pharmacists made visceral connections to seeing adults who could have been *‘my dad, aunty or uncle’* or themselves *‘a patient of my age’*. This connection *‘hit hard’* and generated a *‘sense of dread’*. The PICU pharmacist noted that they were *‘affected emotionally by the sight of children in PICU’.* Initially, there were daily team-based debriefs, but it became apparent that external *‘professional support’* was needed (AQ14). Despite initial reticence about being sufficiently *‘worthy’* of support and scepticism about the psychological offer (AQ15), their engagement with the CPs was described as *‘amazing’* and *‘game-changing’* (AQ16). They all welcomed working with the CPs.

#### Engaging with compassion

The CFT model resonated well with the pharmacists as it was *‘validated’* and *‘wasn’t airy-fairy [but] grounded’*. All pharmacists talked of the *‘three bubbles of soothe, threat and drive’* and noted how helpful it was *‘to put your feelings in each little globe… see how decompensated our lives were’* and to be told *‘it’s ok to feel like you’re feeling’.* They could visualise how disproportionately large the threat and drive bubbles were compared with their soothe bubble (Q17) and to understand what constituted threat and drive (AQ18). Soothe techniques such as *‘meditation, mindfulness, exercise’* helped calm them (AQ19). The pharmacists agreed *‘that [the CPs] had definitely helped us mentally survive the situation’.* Soothing was a short and longer-term investment (AQ20); although not all eligible pharmacists did engage (AQ21).

### Lessons learned

As a result of both their experiences of working in A-ICU or PICU, the pharmacists had learned three main lessons.

#### You’re part of a wider team

Their experiences reinforced the strength of the connection with each other and their awareness they were part of *‘an amazing team’* and triggered a deeper sense of being an authentic part of a wider (A-ICU/PICU) team and that their colleagues, from other disciplines, respected their contributions (AQ22).

#### You’re not indestructible

The *‘huge impact on mental health’* shifted their perceptions of themselves from feeling *‘resilient’* and *‘reserved about sharing their feelings’* to a realisation that they were *‘not indestructible’* but that even in *‘horrendous circumstances, we can prevail*’ (AQ23) and it *‘helps to talk about things’.*

#### You’re better with compassion

Embracing a compassionate outlook impacted their personal and professional lives at the height of COVID-19 and in the months afterwards. Some pharmacists reported being more self-aware and continued to use ‘soothe’ methods (AQ24) Most explained how compassion-informed conversations (AQ25) were now more accepted within their professional lives (AQ26).

## Discussion

The main finding of the study was that the fluidity of the pharmacists’ practice was unsettled as they prepared for and dealt with the factors that threatened them. They were driven to seek skilled support from the PICU clinical psychologists. This support helped them understand how decompensated their lives had become. By adopting a more open, compassionate approach to themselves, their work, and their colleagues, the pharmacists were able to promote and sustain their own well-being and to optimally fulfil their roles and to deliver effective care. The lessons learned from their experiences shaped their mindsets and practice, resulting in a more open, compassionate approach to their work and colleagues.

### Strengths and limitations

A core strength of this study is the use of PE interviews. Using PE meant that the participants were able to share images or objects that illustrated and illuminated their experiences in a meaningful and visual way, adding depth to the words they shared. Also, since their images were the starting points for the stories they shared, they had additional control over their stories. Despite the strengths of the research design, the small sample size may limit the study, although this is offset by the in-depth nature of the interviews. Transferability of the findings may be limited as the setting (stand-alone paediatric tertiary hospital) and situation (two episodes within the COVID-19 pandemic) were unique. The study does not represent the experiences of pharmacists who did not choose to engage with the support offered by the CPs.

### Context of other literature

The discussion is framed by the three affect systems of the CFT model: threat defence, drive and soothe/contentment.^[13]^

Adopting a compassionate mindset and being able to reflect on their own and others’ experiences, feelings and responses helped to support their resilience and allowed the pharmacists to grow personally and develop deeper professional relationships. The orientation to compassion helped to cultivate ‘a sense of safeness’^[25]^ and supported them to reflect and make the changes needed to sustain and optimise their practice. Reflection and its role within the pharmacy workforce were evident pre-pandemic^[26]^ when it was noted that ‘reflections are often the foundation of [pharmacists’] resilience’ (p1186).^[27]^ Pharmacists had a pivotal role in delivering effective healthcare in situations of uncertainty^[3, 4]^ as reported in other studies during the pandemic.

The pharmacists’ understanding of their experiences and feelings, regardless of whether they had been working in A-ICU or PICU, became meaningful through their exploration of the three affect systems – ‘threat defence’, ‘drive’ and ‘soothe and contentment’.

The pharmacists’ sense of threat was activated during the preparation phase but became elevated throughout the time that they were actively involved in their new roles. Threats experienced by the pharmacists resonated with those reported by other healthcare staff, primarily from adult settings, for example, concerns about becoming infected/infecting others, pressures of caring for patients, working in difficult circumstances, uncertainty, changes to practice, increased workloads and managing drug shortages.^[4, 12, 28–30]^ Threats also included the fracturing of existing social structures as people shifted teams, and greater social and physical isolation from those people who typically provided support and emotional infrastructure. As seen with other studies, the threats and the ensuing chaos such as disruption to usual rhythms and changes to practice were challenging.^[5,22]^

The impact on the pharmacists’ well-being was evident and, as seen in other professionals, at times they experienced disorientation and were overwhelmed by what they witnessed^[22]^ in A-ICU and PICU. Other studies on the influence of COVID-19 on pharmacists and other health professionals, in a variety of settings, have shown impacts on well-being such as waking with worry,^[5]^ and fears and issues associated with PPE.^[6,31,32]^ Negative feelings such as anxiety, frustration and a sense of discouragement^[4]^ have been reported, similar to those experienced by the pharmacists in this study. However, their situation was atypical as they gained access to skilled and tailored psychological support considered essential for pharmacists and other healthcare staff^[6,33]^; many healthcare staff during the pandemic globally did not gain this support.

Drive can be a positive force but when it becomes too demanding it can trigger the threat system.^[25]^ The pharmacists characterised themselves as ‘perfectionists’ whose usual goal was to achieve medicine optimisation using their knowledge base and skills in situational analysis. Pre-pandemic they were able to achieve this with confidence; the pandemic unsettled their confidence, as seen in other healthcare staff,^[22,34]^ and drove them to seek information, learn more and be as prepared as possible. Often this information was not available and adaptation was challenging, particularly considering the role critical care pharmacists play in conceptualising, appraising and drafting treatment protocols.^[12]^

The soothing techniques helped address how decompensated their lives had become. These techniques included mindfulness, meditation, exercise and taking time out; these techniques helped to manage the emotional toll. This proactive approach to self-care is not always considered or addressed in the literature addressing the impact of COVID-19 on healthcare staff. However, the importance of practising self-care is seen as important in promoting and prioritising the mental health/well-being of healthcare staff.^[35]^ Self-compassion and mindfulness have been shown to be improved as a result of compassion-related interventions.^[36]^

### Implications

This study shows that resilience and well-being can be supported, even in times of extreme crisis, through the guidance offered using a framework based on CFT. Before the pandemic there were already calls for promoting workplace resilience to promote pharmacists’ well-being.^[37]^ Adopting a proactive approach to self-care has the potential to improve the well-being of pharmacists across a range of settings and could have additional benefits such as promoting retention of staff. Future research should address the impact of compassion-based interventions on the well-being of pharmacists. The adoption of photo-elicitation and other visual-based approaches have merit and potential for future qualitative pharmacy-related research.

## Conclusion

The fluidity of the paediatric pharmacists’ practice was unsettled by the threats associated with the admission of adult COVID-19 patients requiring intensive care in a stand-alone paediatric tertiary hospital. Their shift in role into either A-ICU or PICU challenged their confidence and practice as did having to deal with the threats associated with COVID-19. However, gaining skilled support and drawing on compassion-focused resources helped to support their resilience, sustain and optimise their practice and deepen professional relationships. CFT to support the well-being of pharmacists is a novel contribution to the field.

## Supplementary Material

riac074_suppl_Supplementary_File_S1

riac074_suppl_Supplementary_File_S2

## Data Availability

The data underlying this article cannot be shared publicly due to an ‘exceptional’ condition imposed by the Ethics Committee to ensure the anonymity of the participants; the target population was small and from one hospital and the context unique. The Ethics Committee’s condition extends to the sharing of the raw, anonymised data; it cannot be shared. Please also note, a further condition of the Ethics Committee was that the pre-submission version of the paper should be shared with a member of the wider clinical-academic pharmacy community at the hospital for review to ensure that the author had ensured anonymity.

## Data Access Statement

The author had complete access to the study data (from data generation, data analysis and synthesis). This access is ongoing.
