# Supplementary material for: Unsettling the fluidity of practice and dealing with threat: the experiences of paediatric pharmacists in response to the admission of adult COVID-19 patients requiring intensive care in a paediatric tertiary hospital
Source: Int J Pharm Pract. 2022 Oct 28:riac074. doi: 10.1093/ijpp/riac074 (PMC9620377; doi:10.1093/ijpp/riac074)
Supplement: riac074_suppl_Supplementary_File_S1 [file riac074_suppl_supplementary_file_s1.pdf]

Interview outline

Pharmacy PICU and A-ICU Pandemic Study

Opening

- Discussing study, opportunity to ask questions, checking understanding, decision to participate/decline.
- Reminder of opportunity to pause, stop or withdraw.
- Verbal consent .

Question 1

Please tell me about this image and why you chose it.
Prompts will be used as appropriate (e.g. "please tell me more", "why do think your felt that"?, "what else was happening"?).

Other questions

These questions won't necessarily all be asked or asked in this order and will be used to supplement the image-based responses and/or used in those interviews where the interviewee chooses not to share an image.

What were the challenges (professional, emotional etc.) you experienced as a pharmacist/psychologist during the period in question?

What was the personal/professional impact on you at the time? Are any of these impacts ongoing?

What do think of the 'formal' support you received/gave during this time? Did you turn to other sources of support as well?

How did this support help you navigate that period of time?

What, if any growth or learning came out of your experiences?

Have your experiences influenced how you think about your future role?

Closing

- Thank them for their time, check they are OK.
- Check if they have further questions.
- Sending them the 'Thank you and helpful info' sheet.

v1. 23rd February 2021
Ethics ref ETH2021-0077
