# Supplementary material for: Unsettling the fluidity of practice and dealing with threat: the experiences of paediatric pharmacists in response to the admission of adult COVID-19 patients requiring intensive care in a paediatric tertiary hospital
Source: Int J Pharm Pract. 2022 Oct 28:riac074. doi: 10.1093/ijpp/riac074 (PMC9620377; doi:10.1093/ijpp/riac074)
Supplement: riac074_suppl_Supplementary_File_S2 [file riac074_suppl_supplementary_file_s2.pdf]

Pharmacy PICU and A-ICU Pandemic Study

[Name of participant]
[Date of participation]

Thank you so much for taking part in the study and giving up some of your time and reflecting on some of your experiences of working on PICU or A-ICU or providing support.

If taking part in the study has brought back difficult memories or feelings, support is available to you. If you a pharmacist, support is available from the clinical psychologists. If you are a clinical psychologist, the arrangement is for you to take this to your regular clinical supervision session.

Your participation has made an invaluable contribution.

For any further questions about the study please contact [Name of researcher; email of researcher]

v1. 23rd February 2021
Ethics ref ETH2021-0077
